# Continuous darkness is associated with improved growth performance during a high‐temperature period and sex‐specific modulation of early gonadal development in juvenile yellowtail (*Seriola quinqueradiata*)

**DOI:** 10.1111/jfb.70617

**Published:** 2026-08-14

**Authors:** Yuya Hasegawa, Kiko Shibata, Taiga Abe, Takurou Hotta

**Affiliations:** ^1^ Fisheries Technology Institute, Japan Fisheries Research and Education Agency (FRA) Nagasaki Japan

**Keywords:** continuous darkness, germ cells, gonadal development, meiosis, photoperiod, yellowtail

## Abstract

Selective breeding of yellowtail (*Seriola quinqueradiata*) is expanding in Japan, increasing the demand for practical reproductive control tools in commercial aquaculture to reduce genetic risks from escapees. Slowing early gonadal development through environmental manipulation may support future sterilization strategies, particularly when combined with other techniques. This study aimed to examine somatic growth and early gonadal development in juvenile yellowtail reared under continuous darkness (0L:24D). Juveniles were reared under 0L:24D or a 12L:12D photoperiod (control) and sampled at Weeks 0, 2, 4, 6, 8 and 10. Growth was recorded at each time point, and gonads were examined by histology and immunohistochemistry to assess gonadal sex differentiation, germ‐cell proliferation and onset of meiosis. Water temperature increased similarly in both groups and exceeded 28°C from Week 6 onward. Despite identical feeding rations, fish reared under 0L:24D were significantly larger at Weeks 8 and 10 than controls. Gonadal sex differentiation progressed normally in both groups, and germ‐cell proliferative activity did not differ between groups. SYCP3‐positive germ cells were not detected at Week 6 under 0L:24D, whereas the proportion of SYCP3‐positive germ cells at Weeks 8 to 10 was comparable to that in controls, suggesting a possible transient difference in the initial appearance of SYCP3‐positive germ cells. In females, fish reared under 0L:24D showed lower area fraction and diameter of chromatin nucleolus‐ and perinucleolus‐stage (CN/PN) oocytes at Weeks 8 and 10, suggesting reduced development of CN/PN‐stage oocytes. These results suggest that, under the present single‐tank experimental conditions, rearing under continuous darkness was associated with improved growth performance during the high‐temperature period and sex‐specific changes in early gonadal development in juvenile yellowtail.

## 1 INTRODUCTION

Yellowtail (*Seriola quinqueradiata*) is one of the most important fish species in marine aquaculture in Japan, and selective breeding for traits such as rapid growth has advanced in recent years (Nakada, 2008; Noda et al., 2017; Ozaki et al., 2013). As improved strains are increasingly used, there is growing concern that cultured fish may escape and have genetic impacts on wild populations (Atalah & Sanchez‐Jerez, 2020; Glover et al., 2017). To mitigate these risks, practical sterilization techniques suitable for commercial aquaculture are needed (Piferrer et al., 2009). However, triploid induction and genome editing generally require manipulation at the fertilized‐egg stage and may involve technical, regulatory or societal constraints (Gratacap et al., 2019; Piferrer et al., 2009; Robinson et al., 2024; Roy et al., 2022). Therefore, simpler and more practical alternatives are needed. One possible direction is to regulate early gonadal development during the juvenile stage through environmental conditions. In other words, developing reproductive control technologies requires not only evaluating sterilization itself but also understanding when and how germ cells respond to external environmental factors during development.

In teleosts, primordial germ cells migrate to and colonize the gonadal primordium. After colonization, germ cells undergo a proliferative phase, followed by the onset of meiosis, and subsequently differentiate into oocytes or spermatocytes (Nishimura & Tanaka, 2014; Robles et al., 2017; Schulz et al., 2010). In this process, gonadal sex differentiation, germ‐cell proliferation, the onset of meiosis and early oocyte development are major developmental events that characterize early gonadal development. Because germ cells cease mitotic proliferation upon entering meiosis, sterility may result if proliferating germ cells are depleted before the onset of meiosis (Schulz et al., 2010). Clarifying which stages of this developmental process are most responsive to external environmental factors is important not only for understanding early gonadal development but also for designing future reproductive control strategies.

Among externally controllable factors in aquaculture, environmental conditions such as photoperiod and water temperature are particularly practical because they can be manipulated without handling individual fish. In many cultured fish species, the timing of sexual maturation can be adjusted by manipulating photoperiod and water temperature and photoperiod control is also used in yellowtail broodstock operations (Bromage et al., 2001; Mushiake et al., 1998). Photoperiodic effects are known to be associated with regulation of reproductive endocrine pathways, including melatonin signalling and the hypothalamus–pituitary–gonad (HPG) axis and may also affect gonadotropin (GTH) secretion (Bromage et al., 2001; Falcón et al., 2010; Maitra & Hasan, 2016; Migaud et al., 2010). In the closely related greater amberjack, long‐day treatment affects plasma follicle‐stimulating hormone (FSH) release and pituitary *fshb/lhb* expression in previtellogenic fish (Nyuji et al., 2018). In addition, temperature and photoperiod affect sex determination in California grunion (Brown et al., 2014). In European sea bass, artificial photoperiod alters pituitary gonadtropin‐releasing hormone (GnRH) profiles and plasma luteinizing hormone (LH) profiles during the testicular differentiation and growth period in juveniles that were initially undifferentiated at 4 months of age (Rodríguez et al., 2004). Together, these findings suggest that the light environment may influence not only maturation but also sex determination or differentiation‐related processes and reproductive endocrine activity during juvenile development. However, most of these studies have mainly focused on reproductive endocrine responses and studies directly evaluating stage‐specific changes in germ‐cell development within the gonad remain limited. Therefore, how photoperiod is associated with specific stages of early gonadal development during the juvenile period, including gonadal sex differentiation, germ‐cell proliferation, the onset of meiosis and early oocyte development, remains insufficiently understood.

Continuous darkness (0L:24D) is an extreme but simple photoperiod manipulation that removes diel light cues and can be useful for detecting developmental responses to the light environment. Accordingly, evaluating early gonadal development under this condition may help identify developmental stages that are responsive to the light environment.

As described above, it remains unclear whether continuous darkness affects early gonadal development in juvenile yellowtail. This study aimed to identify which developmental stages are sensitive to continuous darkness. We applied continuous darkness and examined its effects on gonadal sex differentiation, germ‐cell proliferation, the onset of meiosis and **e**arly oocyte development using histological and immunohistochemical analyses.

## 2 MATERIALS AND METHODS

### 2.1 Fish

Juvenile yellowtail were produced by artificial fertilization at the Goto Field Station (Fisheries Technology Institute, Nagasaki, Japan) (hatch date: 24 April 2025) and reared until the start of the experiment (16 June 2025). The experimental fish were derived from multiple families. At the start of the experiment, fish were 50 days after hatching (dah) and had a mean body weight (BW) of 2.8 ± 0.4 g and a mean total length (TL) of 6.6 ± 0.3 cm (*n* = 20) (Figure 1b,c). Fish were maintained in 500 L tanks supplied with continuous flow‐through sand‐filtered and UV‐sterilized seawater. Water temperature was maintained at 22°C until 23 June and thereafter followed seasonal ambient conditions. During the larval stage, fish were fed rotifers (*Brachionus plicatilis*) and *Artemia nauplii*, after which they were switched to a commercially formulated diet from 30 dah. Fish were fed to satiation during the pre‐experimental rearing period.

**FIGURE 1:**
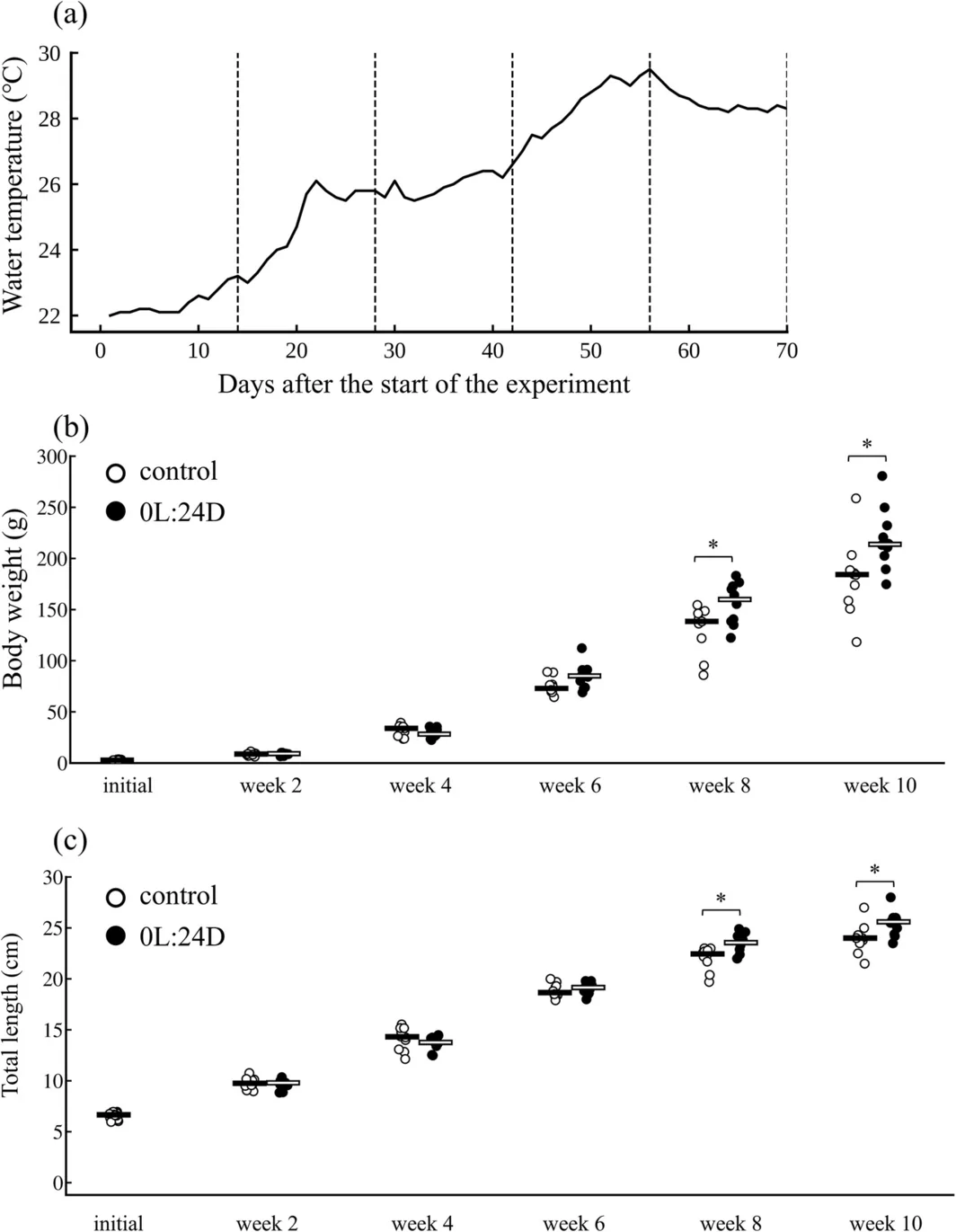
Water temperature profile and growth performance. (a) Daily water temperature (°C) during the experimental period. Vertical dashed lines indicate sampling Weeks (2, 4, 6, 8 and 10 Weeks after the start of the experiment). (b) Body weight and (c) total length measured at each sampling point (initial and Weeks 2, 4, 6, 8 and 10). Open and black circles indicate the control and 0L:24D group, respectively. Initial data were collected at the start of the experiment and are shown as baseline values. Each circle represents an individual fish, and thick horizontal bars denote medians.

### 2.2 Experimental design

Fish at 50 dah were randomly assigned to two photoperiod treatments: continuous darkness (0L:24D) and a 12L:12D photoperiod (control). Each photoperiod treatment was conducted in a single 500‐L tank, and tank‐level replication was not available. To achieve 0L:24D conditions, the tank was fully covered with light‐blocking sheets and all potential light leaks were sealed. Light intensity in the 0L:24D tank was confirmed to be 0 lux using a light metre. Fish in the control group were maintained under white fluorescent illumination (1000 lux at the water surface). Feeding in the 0L:24D group was conducted through a slit/opening in the cover to minimize light exposure. Water temperature in both groups was recorded daily. During the experimental period, fish were fed a fixed daily ration (quantitative feeding) and the same ration was provided to both groups. No visible uneaten feed was observed in the control tank after feeding. However, uneaten feed in the 0L:24D tank could not be reliably monitored because the tank was kept covered to maintain continuous darkness.

### 2.3 Sampling

At Weeks 0, 2, 4, 6, 8 and 10, fish were netted and anaesthetized with 2‐phenoxyethanol (1:2000 dilution in seawater; 0.05% v/v) prior to sampling. Fish were then euthanized by transection of the medulla oblongata, followed immediately by exsanguination before gonad dissection and fixation. BW and TL were measured. At Weeks 0 and 2, whole fish were fixed. From Week 4 onward, gonads were dissected and fixed, and fins were collected for genetic sex determination. Fin samples were transferred to ethanol and kept at 4°C overnight for solvent replacement, then stored at −20°C. Gonads sampled at Weeks 0 to 4 were fixed in Davidson's solution. At Weeks 6, 8 and 10, gonads were fixed in Bouin's solution overnight at room temperature. In parallel, separate gonad samples were fixed overnight at 4°C in 4% paraformaldehyde (PFA) prepared in phosphate‐buffered saline (PBS). For samples from the initial time point to Week 4, whole fish or very small dissected gonads were fixed and could not be divided for separate H&E staining and immunohistochemistry; therefore, these samples were fixed in Davidson's solution, which was compatible with both analyses in preliminary trials. From Week 6 onward, gonads were large enough to be divided, allowing the use of analysis‐specific fixatives to improve staining quality: Bouin's solution for H&E staining and 4% PFA for immunohistochemistry.

All experimental procedures were approved by the Institutional Animal Care and Use Committee of the Fisheries Technology Institute, Japan Fisheries Research and Education Agency, and were conducted in compliance with relevant ethical standards and guidelines.

### 2.4 Genetic sex determination (PCR–RFLP)

Genetic sex of yellowtail was determined by polymerase chain reaction–restriction fragment length polymorphism (PCR–RFLP) at the *Hsd‐17b1 locus* as previously described (Koyama et al., 2019), in which the W allele is cleaved by BsaWI whereas the Z allele is not. Genomic DNA was prepared from fin clips using Tail Lysis Buffer (Nacalai Tesque, Japan) containing 0.5 mg/mL Proteinase K (MACHEREY‐NAGEL, Germany). The lysate was diluted 1:200 with nuclease‐free water and used as the PCR template. The Hsd17b1 region was amplified using primers GCCTGCACAAAGACACCTTGG (forward) and ACTGGACCACACTCAATGAGAC (reverse) with KOD One PCR Master Mix (TOYOBO, Japan). PCR products were digested with BsaWI (New England Biolabs, USA) at 60°C for 1 h and separated by 2% agarose gel electrophoresis. Individuals were genotyped based on banding patterns (ZZ: uncut; ZW: both uncut and cut fragments), and genetic sex was assigned accordingly. Genetic sex was compared with the histological gonadal phenotype to determine whether 0L:24D caused a mismatch between genetic sex and gonadal phenotype.

### 2.5 Histological analysis

Samples fixed in Davidson's solution or PFA were rinsed three times in PBS, whereas Bouin‐fixed samples were processed without PBS rinsing. All samples were dehydrated through a graded ethanol series, cleared in methyl benzoate (FUJIFILM Wako Pure Chemical Corporation, Japan) and UI‐zol (UI KASEI Co., Ltd., Japan) and embedded in paraffin. Sections were cut at 5 μm using a microtome. For general morphology, sections were stained with haematoxylin and eosin (H&E) for light microscopic evaluation.

For histological classification, oogonia were identified as small germ cells with a high nuclear‐to‐cytoplasmic ratio. Chromatin nucleolus (CN)‐stage oocytes were identified as early oocytes with an enlarged nucleus and chromatin/nucleolar structures, whereas perinucleolus (PN)‐stage oocytes were identified by the presence of multiple nucleoli arranged along the nuclear periphery. In males, spermatogonia were identified as relatively large germ cells with round nuclei and a high nuclear‐to‐cytoplasmic ratio. Spermatocyte‐like cells were identified as smaller germ cells within cyst‐like structures, showing increased nuclear density and more condensed chromatin than spermatogonia.

### 2.6 Immunohistochemistry

SYCP3 was used as a marker of germ cells that had entered meiosis because it is a structural component of the synaptonemal complex and is expressed in meiotically dividing germ cells in fish (Iwai et al., 2006). Paraffin sections were deparaffinized, rehydrated and subjected to antigen retrieval using Immunosaver (Nisshin EM, Japan) according to the manufacturer's instructions. Non‐specific binding was blocked with 5% (w/v) bovine serum albumin (BSA; Nacalai Tesque, Japan) for 1 h at room temperature. Sections were incubated overnight at 4°C with primary antibodies diluted 1:500 in blocking buffer. For double immunofluorescence, sections were co‐incubated with either mouse anti‐DDX4/Vasa (Thermo Fisher Scientific, USA; MA5‐56910) and rabbit anti‐SYCP3 (Abcam, UK; ab15093), or rabbit anti‐Vasa (GeneTex, USA; GTX128306) and mouse anti‐PCNA (Abcam, UK; ab29). After washing, sections were incubated with Alexa Fluor 594–conjugated goat anti‐rabbit IgG (H + L) (Thermo Fisher Scientific, USA; A‐11012) and Alexa Fluor 488–conjugated goat anti‐mouse IgG (H + L) (Thermo Fisher Scientific, USA; A‐11001) at 1:500 for 60 min at room temperature in the dark. Nuclei were counterstained with DAPI (Thermo Fisher Scientific, USA; 62,248), and slides were mounted with VECTASHIELD Antifade Mounting Medium (Vector Laboratories, USA). Negative controls included normal rabbit IgG and normal mouse IgG (isotype controls) in place of the primary antibodies at the same concentration, followed by the corresponding secondary antibodies (Figure S2). To provide Supporting Information for the commercial antibodies used in this study, peer‐reviewed published applications of the same antibodies with the same catalogue numbers are summarized in Table S1. For SYCP3, amino‐acid sequences from yellowtail and other vertebrates, including species in which the same anti‐SYCP3 antibody has been used in previous studies, were aligned to assess conservation of the corresponding C‐terminal region (Figure S3).

### 2.7 Image acquisition and quantitative analysis

Images were acquired using a Nikon ECLIPSE Ni microscope equipped with a Nikon Digital Sight 10 camera (Nikon, Japan), using identical acquisition settings within each staining batch. Quantitative analysis was performed using ImageJ (v1.54p). For immunostaining, five microscopic fields were randomly selected from each section (*n* = 5). Vasa‐positive germ cells were counted in each field. PCNA‐positive cells were defined as Vasa‐positive germ cells showing a distinct nuclear signal above the local background; the proportion of PCNA‐positive cells was calculated as (number of Vasa^+^PCNA^+^ cells/number of Vasa^+^ cells) × 100. SYCP3‐positive cells were quantified in the same manner, defining SYCP3 positivity as a specific intranuclear signal above background within Vasa‐positive cells. The time points for SYCP3 quantification were selected based on the sex‐specific timing of gonadal development observed in H&E‐stained sections. In females, SYCP3‐positive cells were quantified at Weeks 4 and 6 to evaluate the onset of meiosis before CN/PN‐stage oocytes became clearly developed. In males, SYCP3‐positive cells were quantified at Weeks 6, 8 and 10 because meiotic germ cells appeared later and were followed during this period.

Oocyte area analysis was performed using H&E‐stained sections. From each section, multiple microscopic fields were randomly selected (*n* = 5). Within each field, oocytes were classified into CN and PN stages based on the morphological criteria described above, and the area of each CN/PN‐stage oocyte was measured. The total germ‐cell area in each field was determined on H&E‐stained sections by manual delineation of regions occupied by germ cells, based on histological features such as high nuclear density and characteristic gonadal germ‐cell morphology. Non‐germ‐cell components, including stromal tissue, gonadal somatic cells, blood vessels and blood cells, were excluded from this area. The CN/PN oocyte area fraction was calculated for each field as the total area of CN/PN oocytes divided by the total germ‐cell area and the mean value across fields within a section was used as the representative value for each individual. This index represents the relative occupancy of CN‐ and PN‐stage oocytes within the germ‐cell compartment and was used as a tissue‐level indicator of the appearance and early growth of primary growth oocytes. In addition, oocyte diameter was calculated from the measured oocyte area.

### 2.8 Statistical analysis

Statistical analyses were performed in R (version 4.4.0). Because each photoperiod condition consisted of a single tank, the tank was the experimental unit for evaluating the photoperiod treatment. Therefore, statistical comparisons based on individual fish were interpreted as exploratory individual‐level comparisons rather than replicated tank‐level tests of photoperiod effects. For each sampling week, differences between the two groups were assessed using Welch's *t*‐test. To account for multiple testing across sampling weeks, *p* values were adjusted within each endpoint using Holm's method, with the number of comparisons defined by the number of weeks included for that endpoint. Differences between groups were considered statistically significant at adjusted *p* < 0.05.

## 3 RESULTS

### 3.1 Water temperature profile during the trial

Water temperature increased progressively over the rearing period and exceeded 28°C at 48 days after the start of the experiment. There was no detectable difference in water temperature between the control and 0L:24D groups throughout the experiment (Figure 1a).

### 3.2 Growth performance

Changes in BW and TL are shown in Figure 1b,c. Although both groups were fed the same ration, fish reared under 0L:24D exhibited higher BW and TL than controls at Weeks 8 and 10 in exploratory individual‐level comparisons. At Week 8, BW was 131 ± 23 g in the control group and 155 ± 20 g in the 0L:24D group, and TL was 22.0 ± 1.1 cm and 23.4 ± 1.1 cm, respectively. At Week 10, BW was 180 ± 37 g in controls and 219 ± 30 g in 0L:24D fish and TL was 24.0 ± 1.4 cm and 25.4 ± 1.2 cm, respectively (Figure 1b,c).

### 3.3 Gonadal morphology assessed by H&E staining

Representative H&E sections are shown in Figure 2 and Figure S1. At Weeks 0 and 2, gonads remained morphologically undifferentiated in both groups. Morphological gonadal sex differentiation became apparent from Week 4 onward. Across all individuals examined, no mismatch between genetic sex and histological gonadal phenotype was observed, and no abnormal gonads, such as intersex gonads or ovotestes, were detected. The sex‐specific progression of germ‐cell development based on H&E‐stained sections is summarized in Table 1. In females, oogonia were observed from Week 4 onward, and early‐stage oocytes were observed from Week 6 onward. CN/PN‐stage oocytes first became identifiable at Week 8. In males, spermatogonia were predominant from Week 4 onward, and spermatocyte‐like cells first became apparent at Week 6 in controls and at Week 8 in the 0L:24D group.

**FIGURE 2:**
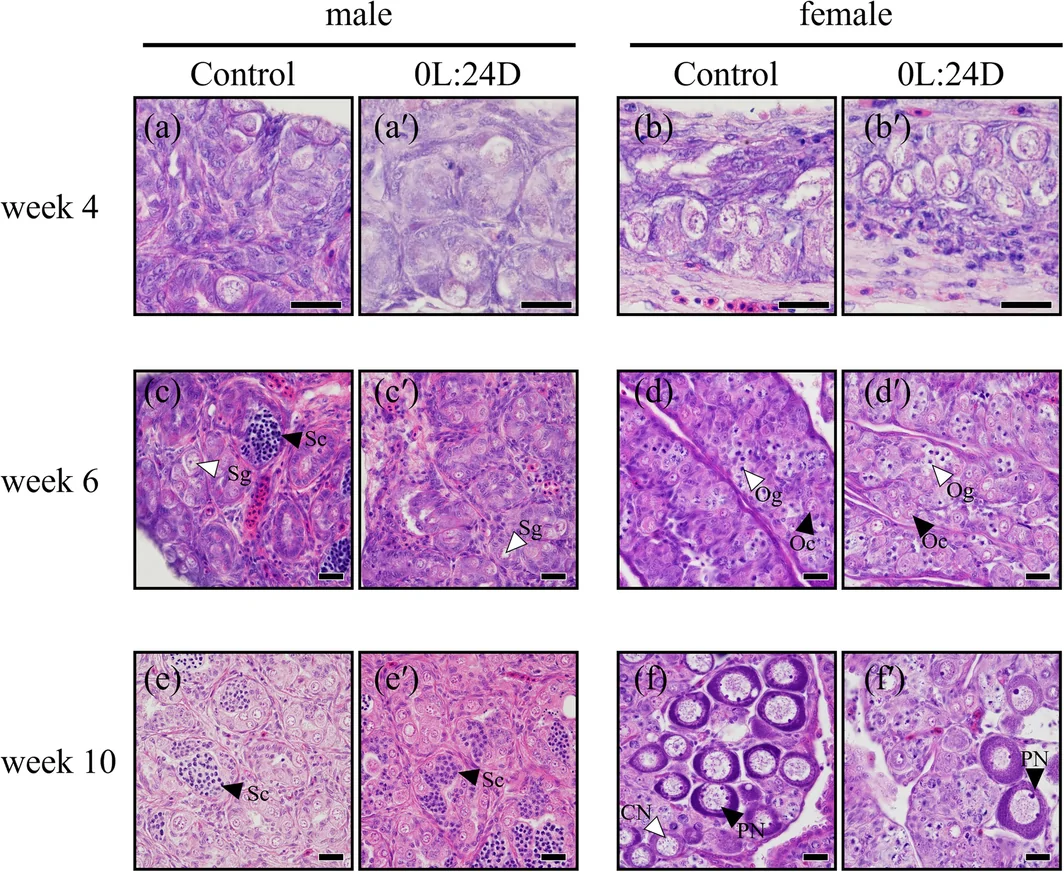
Representative haematoxylin and eosin (H&E)‐stained gonadal sections. Male and female gonads are shown at Week 4 (a, a′, b, b′), Week 6 (c, c′, d, d′) and Week 10 (e, e′, f, f′). Panels show male Control (a, c, e), male 0L:24D (a′, c′, e′), female Control (b, d, f) and female 0L:24D (b′, d′, f′). Arrowheads indicate representative germ‐cell types where clearly identifiable. CN, chromatin nucleolus‐stage oocytes; Oc, early‐stage oocytes; Og, oogonia; PN, perinucleolus‐stage oocytes; Sc, spermatocyte‐like cells; Sg, spermatogonia. Black and white arrowheads were used only to improve visibility against the histological background. Scale bars = 20 μm.

**TABLE 1:** Summary of sex‐specific gonadal development based on haematoxylin and eosin (H&E) staining and time points used for immunohistochemical quantification.

Sampling week	Female gonadal morphology based on H&E staining	Male gonadal morphology based on H&E staining	PCNA‐IHC quantified	SYCP3‐IHC quantified
Week 2	Morphologically undifferentiated	Morphologically undifferentiated	‐	‐
Week 4	Oogonia	spermatogonia	Females and males	Females
Week 6	Early‐stage oocyte	Spermatocyte‐like cell in control group	Females and males	Females and males
Week 8	CN/PN‐stage oocytes	Spermatocyte‐like cell	Females and males	Males
Week 10	CN/PN‐stage oocytes	Spermatocyte‐like cell	‐	Males

*Note*: Gonadal morphology was assessed using H&E‐stained sections. The PCNA‐IHC and SYCP3‐IHC columns indicate the sexes and sampling weeks used for immunohistochemical quantification. A dash indicates that immunohistochemical quantification was not performed at that sampling week. Spermatocyte‐like cells were identified based on H&E‐stained morphology.

Abbreviations: CN, chromatin nucleolus; IHC, immunohistochemistry; PN, perinucleolus.

### 3.4 Proliferative activity assessed by PCNA immunostaining

Representative PCNA and Vasa immunofluorescence images of gonads are shown in Figure 3. The proportion of PCNA‐positive cells among Vasa‐positive germ cells was quantified in females and males at Weeks 4, 6 and 8. No significant differences were detected between the control and 0L:24D groups at any time point in females and males (Figure 5a,b).

**FIGURE 3:**
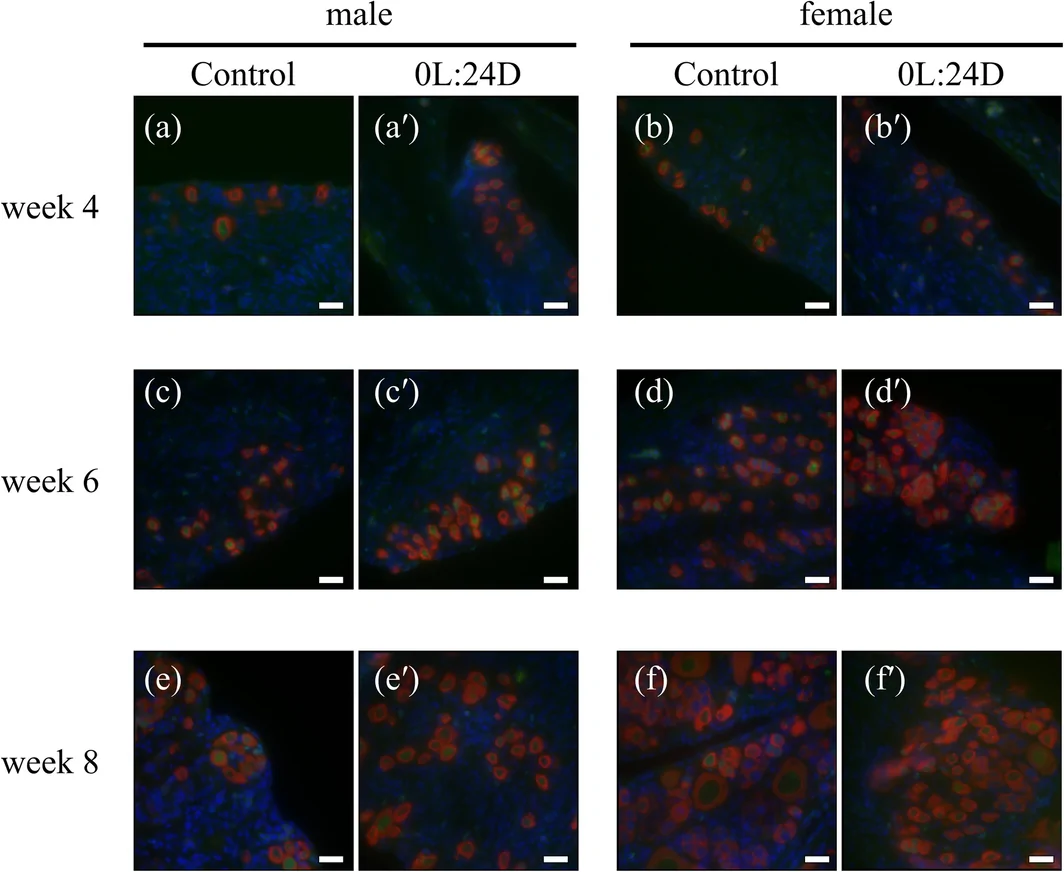
Representative PCNA and Vasa immunofluorescence images of gonads. Gonads from males (left) and females (right) are shown at Week 4 (a–b′), Week 6 (c–d′) and Week 8 (e–f′). Panels: Male Control (a, c, e), male 0L:24D (a′, c′, e′), female Control (b, d, f) and female 0L:24D (b′, d′, f′). PCNA (green), Vasa (red) and nuclei counterstained with DAPI (blue). Scale bars, 20 μm.

### 3.5 SYCP3‐positive germ cells assessed by immunostaining

Representative SYCP3 and Vasa immunofluorescence images of gonads are shown in Figure 4. The proportion of SYCP3‐positive cells among Vasa‐positive germ cells was quantified in females at Weeks 4 and 6 and in males at Weeks 6, 8 and 10; values are presented as percentages (mean ± standard deviation [SD]). In females, SYCP3‐positive cells were not detected at Week 4, and no significant difference was detected between the control and 0L:24D groups at Week 6. In males, the proportion at Week 6 was significantly lower in the 0L:24D group than in controls (control: 3.3 ± 3.2% vs. 0L:24D: 0.0%). At the individual level, SYCP3‐positive germ cells were detected in 5 of 7 control males, whereas no SYCP3‐positive germ cells were detected in any of the 7 males in the 0L:24D group. No significant differences were observed at Weeks 8 and 10 (Figure 5c,d).

**FIGURE 4:**
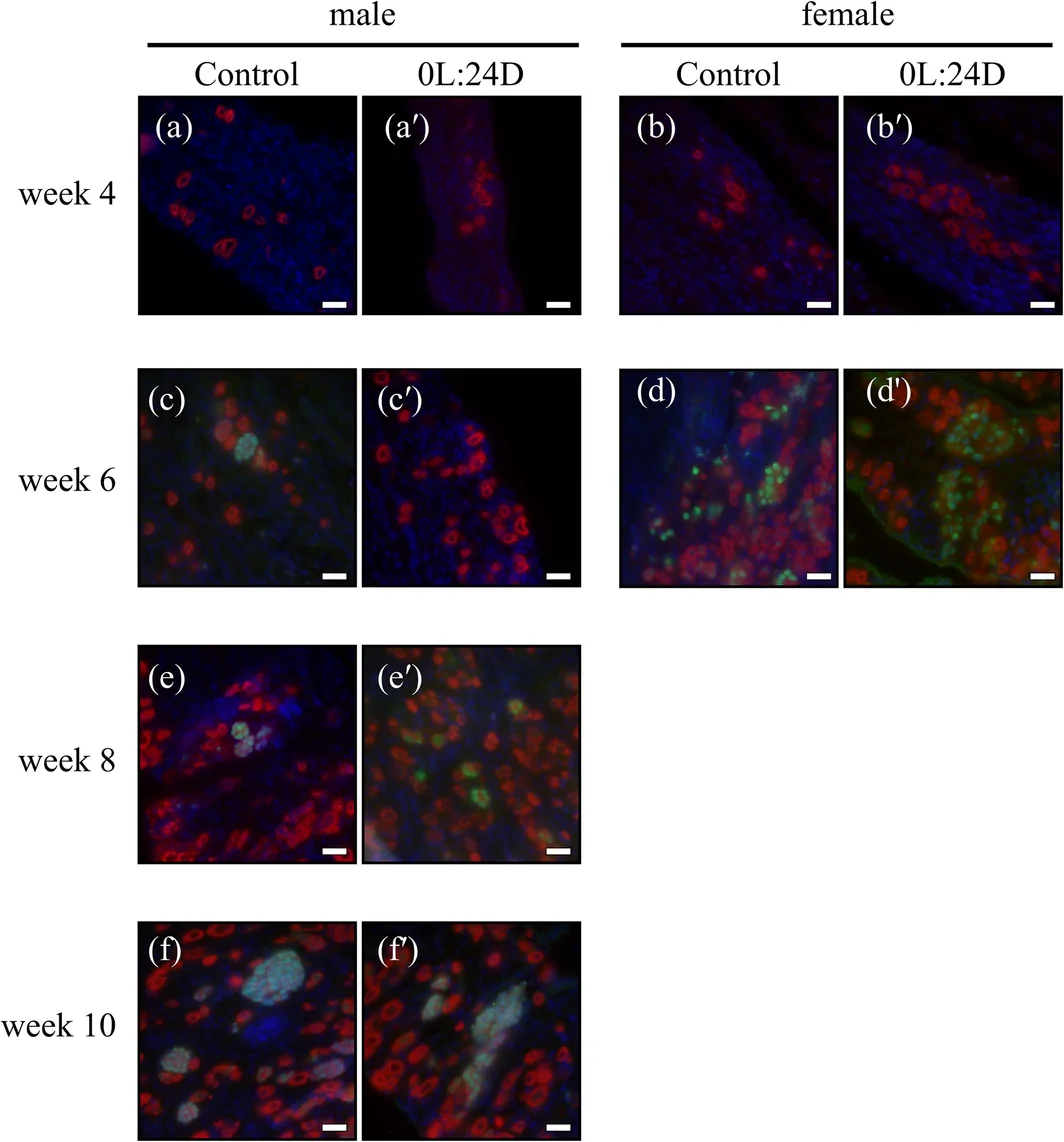
Representative SYCP3 and Vasa immunofluorescence images of gonads. Gonads from males (left) and females (right) are shown at Week 4 (a–b′) and Week 6 (c–d′). Male gonads are additionally shown at Week 8 (e, e′) and Week 10 (f, f′). Panels: Male Control (a, c, e, f), male 0L:24D (a′, c′, e′, f′), female Control (b, d) and female 0L:24D (b′, d′). SYCP3 (green), Vasa (red) and nuclei counterstained with DAPI (blue). Scale bars, 20 μm.

**FIGURE 5:**
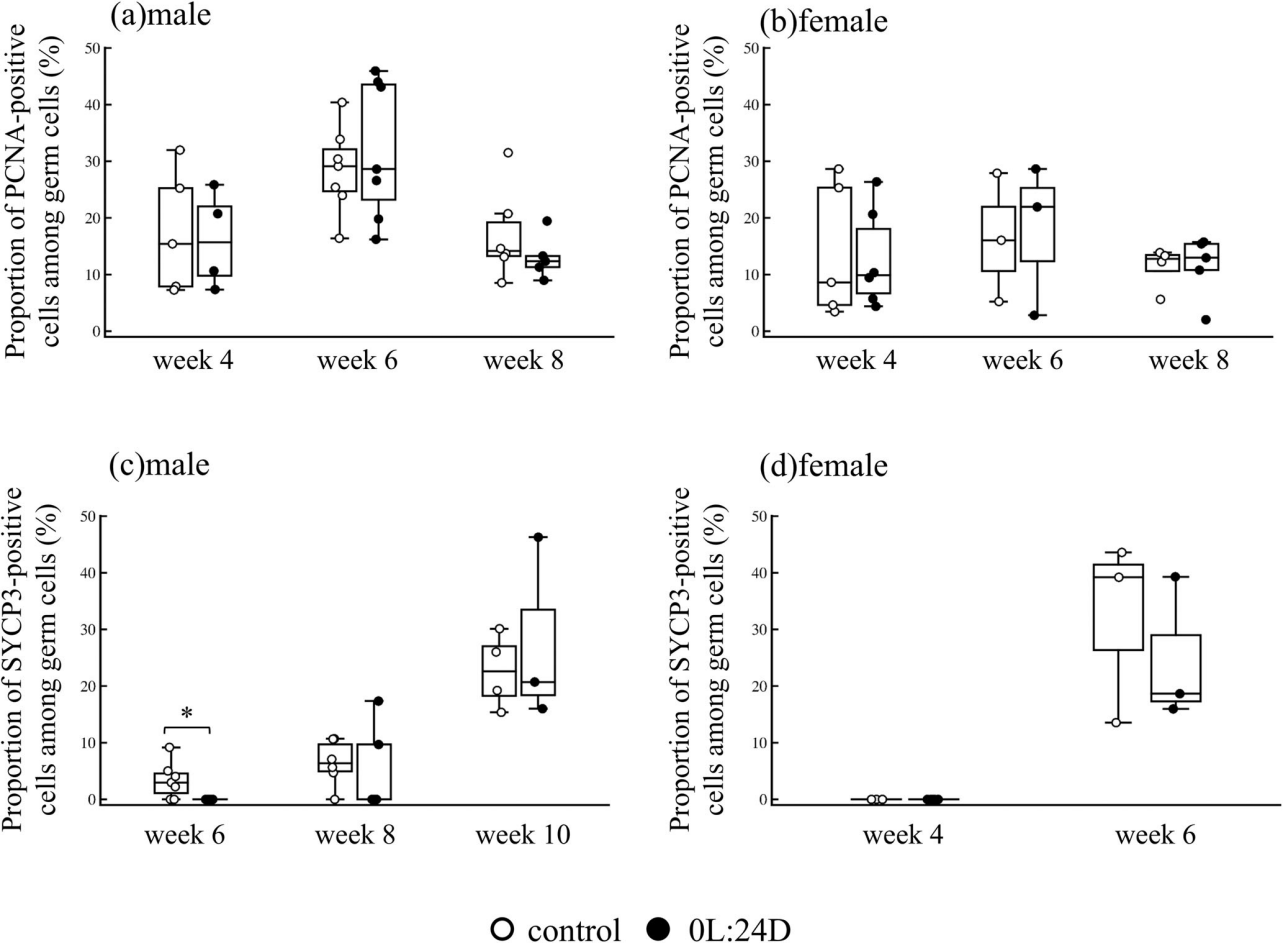
Effects of continuous darkness on PCNA‐positive and SYCP3‐positive germ‐cell fractions. PCNA‐positive fraction at each sampling point in (a) males and (b) females. SYCP3‐positive fraction at each sampling point in (c) males and (d) females. Open and black circles indicate the control and 0L:24D group, respectively. Each circle represents an individual. Boxes indicate the interquartile range with the median as the horizontal line; whiskers indicate the range. Statistical comparisons between treatments at each time point were performed using Welch's *t*‐test, followed by Holm adjustment within each endpoint; *adjusted *p* < 0.05.

### 3.6 Area fraction and diameter of oocytes at CN and PN

The proportion of CN/PN‐stage oocytes within the total germ‐cell area was quantified at Weeks 8 and 10 (Figure 6c,d). The CN/PN oocyte area fraction was significantly lower in the 0L:24D group than in controls. At Week 8, values were 11.7 ± 2.4% in controls and 3.3 ± 3.3% in the 0L:24D group; at Week 10, they were 13.6 ± 7.3% and 1.6 ± 1.2%, respectively (Figure 6c). Oocyte diameter was also significantly lower in the 0L:24D group than in controls. At Week 8, diameters were 34.4 ± 1.9 μm in controls and 26.8 ± 2.0 μm in the 0L:24D group; at Week 10, they were 40.1 ± 1.6 μm and 30.6 ± 2.7 μm, respectively (Figure 6d).

**FIGURE 6:**
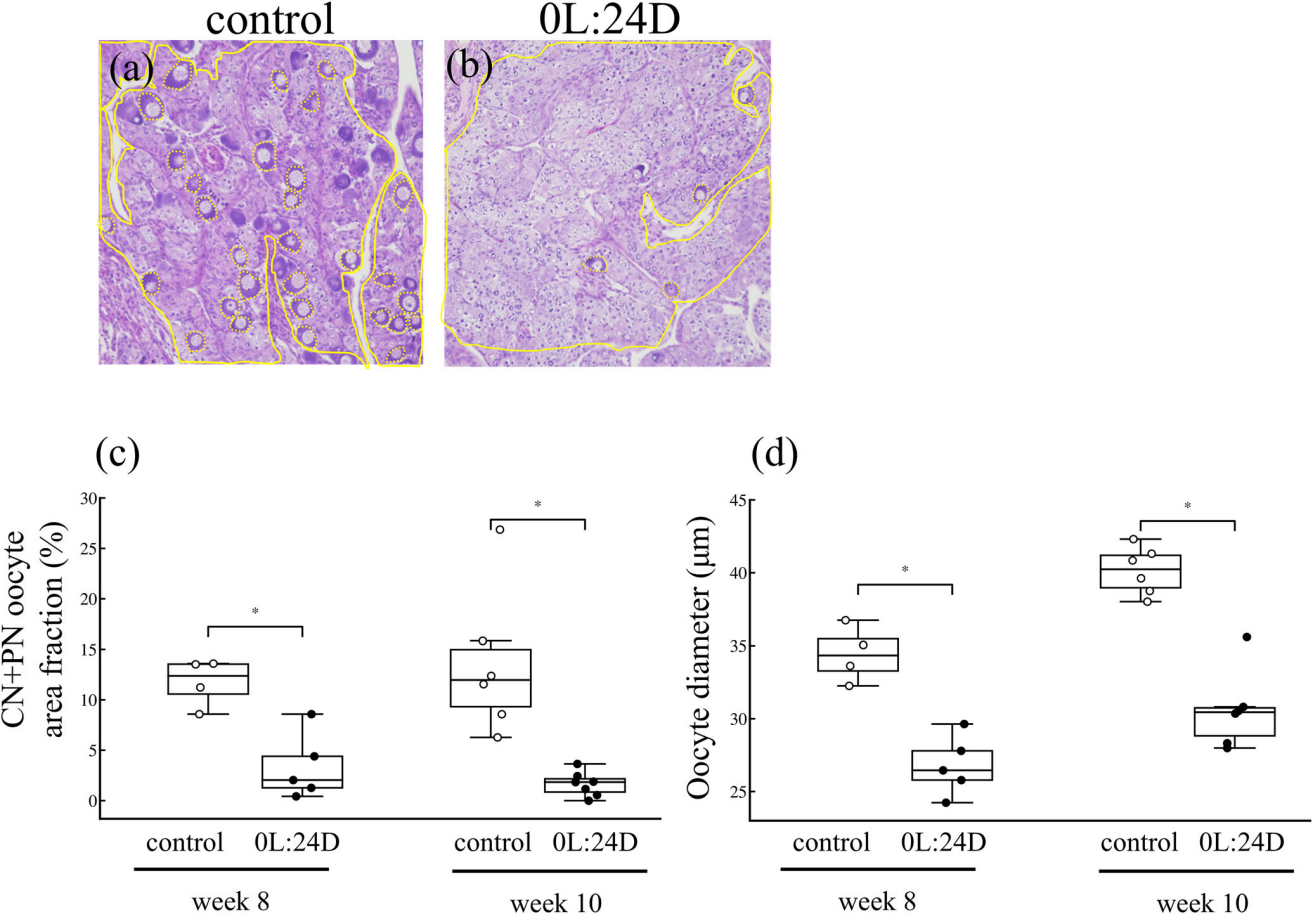
Area fraction and diameter of chromatin nucleolus‐ and perinucleolus‐stage (CN/PN) oocytes (a, b) Representative haematoxylin and eosin (H&E)‐stained ovarian sections illustrating the image‐analysis workflow. The solid yellow outline indicates the total germ‐cell area used as the denominator, and dotted yellow outlines indicate CN‐ and PN‐stage oocytes used as the numerator. (c) CN/PN oocyte area fraction at Weeks 8 and 10 in the control and 0L:24D groups. (d) Oocyte diameter of CN/PN‐stage oocytes at Weeks 8 and 10 in the control and 0L:24D groups. Each circle represents an individual. Boxes indicate the interquartile range with the median as the horizontal line; whiskers indicate the range. Brackets denote pairwise comparisons between groups at each time point using Welch's *t*‐test, followed by Holm adjustment within this endpoint; *adjusted *p* < 0.05.

## 4 DISCUSSION

In this study, we examined how continuous darkness (0L:24D) affects early gonadal development in juvenile yellowtail. We first assessed whether continuous darkness affects growth. From Weeks 2 to 6, BW and TL were similar between groups. However, despite identical rations being provided, the 0L:24D group was significantly larger at Weeks 8 and 10. Water temperature was similar between groups and rose above 28°C after Week 6. In yellowtail, feeding activity may decrease at temperatures above 28°C (Kohbara et al., 2003). In addition, rearing at 30°C can reduce survival and growth, as well as feed and protein utilization (Sotoyama et al., 2018). Thus, the high‐temperature period after Week 6 likely imposed a growth constraint. Because the group difference emerged during this period, continuous darkness may have mitigated heat‐related physiological stress or reduced activity‐related energy expenditure, thereby reducing growth suppression under high‐temperature conditions. However, we did not measure actual feed intake, activity or metabolic/stress indicators, so the underlying mechanism remains unclear.

In addition, to maintain continuous darkness, we did not monitor uneaten feed in the 0L:24D tank. As a result, we could not quantitatively evaluate or compare feed intake between groups. Yellowtail can feed not only during the day but also at night under demand‐feeding conditions, and their feeding behaviour changes with light intensity (Kohbara et al., 2003). In European sea bass, feeding is possible even under dark conditions, suggesting that chemical sensing plays an important role in night feeding (Faucher et al., 2006). Taken together, feeding in the 0L:24D group may have been maintained through senses other than vision.

Our time‐course sampling of gonads from 50 to 120 dah covered stages from before morphological gonadal sex differentiation to the appearance of PN‐stage oocytes in females and spermatocytes in males. Based on H&E staining, the progression of gonadal sex differentiation was similar between groups; no abnormal gonads were observed, and gonadal sex matched genetic sex in all individuals. These observations suggest that continuous darkness, under our experimental conditions, did not disrupt gonadal sex differentiation. In addition, in both sexes, the proportion of PCNA‐positive cells among Vasa‐positive germ cells did not differ between groups, indicating that continuous darkness did not strongly change germ‐cell proliferative activity. Overall, gonadal sex differentiation and germ‐cell proliferation could proceed normally under 0L:24D.

In many teleosts, effects of the light environment are often attributed to endocrine regulation via melatonin (Bromage et al., 2001; Falcón et al., 2010), and even at the pre‐pubertal stage, photoperiod can alter the expression of genes related to the HPG axis (Martins et al., 2015). Thus, even if melatonin signalling or HPG‐axis activity was affected by continuous darkness, such changes may not have been strong enough to disrupt gonadal sex differentiation or germ‐cell proliferation in this study. Alternatively, gonadal sex differentiation and early germ‐cell proliferation may be regulated predominantly by gonad‐intrinsic and/or local factors, with limited dependence on the HPG axis at early stages. It is also possible that these processes might not respond immediately to changes in the HPG axis. In males, the proportion of SYCP3‐positive cells at Week 6 was significantly lower in the 0L:24D group than in controls. Although the absolute frequency of SYCP3‐positive cells was low, their detection in 5 of 7 control males but in none of the 7 males in the 0L:24D group suggests a possible difference in the initial appearance of SYCP3‐positive germ cells under continuous darkness. However, at Weeks 8 and 10, the proportion of SYCP3‐positive cells in the 0L:24D group did not differ from controls. These results suggest that the difference in the initial appearance of SYCP3‐positive germ cells in males under continuous darkness was transient. Because SYCP3‐positive germ cells were used as an indicator of meiotic germ cells, this pattern may reflect a short‐term shift in the timing of meiotic entry. At the same time, the lack of a sustained difference indicates that continuous darkness alone may be insufficient to suppress male reproductive progression over the long term.

In females, both the area fraction and mean diameter of CN/PN oocytes were lower in the 0L:24D group than in controls, suggesting that the appearance and early development of CN/PN oocytes after meiotic entry were reduced under continuous darkness. The mechanism underlying this suppression remains unclear. Photoperiod and melatonin can influence reproductive endocrine pathways in teleosts, including GTH secretion (Maitra & Hasan, 2016; Nyuji et al., 2018). In addition, recent studies have identified cholecystokinin (CCK) as an FSH‐releasing hormone in medaka and as a regulator of GTH secretion in zebrafish, suggesting that hypothalamic regulators other than GnRH may also contribute to FSH regulation in teleosts (Hollander‐Cohen et al., 2024; Uehara et al., 2024). Therefore, future studies targeting endocrine factors such as melatonin, GnRH, CCK, FSH, LH, sex steroids and ovarian signalling pathways will be required to clarify the mechanisms by which continuous darkness affects early oocyte development in yellowtail.

This study has several limitations. First, each photoperiod treatment was conducted in a single tank; thus, potential tank‐specific effects (e.g., subtle differences in rearing environment, feeding conditions or social interactions) cannot be separated from the photoperiod effect. In addition, because the tank, rather than individual fish, is the experimental unit for evaluating the photoperiod treatment, individual fish cannot be treated as independent experimental replicates for treatment‐level inference. Therefore, statistical comparisons based on individual fish should be interpreted as exploratory individual‐level comparisons within this single‐tank experiment, rather than definitive replicated tests of photoperiod effects. The present findings therefore require confirmation with replicated tanks. Second, 0L:24D is an extreme light condition and is not realistic for direct application in sea cages. For practical application, more realistic conditions, such as partial shading and intermediate light levels, should be tested. Third, because the observation period was limited to 120 dah, we could not assess reversibility of the effects, timing of maturation or final reproductive capacity. Fourth, we did not directly measure key factors that may link improved growth performance during the high‐temperature period and changes in early gonadal development, such as feed intake, activity, metabolic rate, stress responses and endocrine dynamics, including HPG‐axis activity. Although both groups received the same ration, actual feed intake in the 0L:24D group could not be quantified because the tank was kept covered to maintain continuous darkness. Therefore, differences in feeding behaviour, feed utilization efficiency, activity or metabolic expenditure may have contributed to the observed growth difference. Finally, direct validation of the antibodies in yellowtail by Western blotting or peptide pre‐absorption was not performed in this study. Therefore, the immunohistochemical results should be interpreted together with the supporting evidence provided by published applications of the same antibodies, conservation of the corresponding SYCP3 region and host‐matched isotype controls. Future studies should quantify actual feed intake and feed conversion efficiency under controlled light conditions. In addition, practical shading conditions should be evaluated using replicated tank designs, and the observation period should be extended. Behavioural, metabolic, stress and endocrine parameters should also be characterized to clarify the relationship between improved growth performance during high‐temperature periods and changes in early gonadal development under different light regimes.

In summary, the present study suggests that, under the present single‐tank experimental conditions, continuous darkness was associated with improved growth performance during the high‐temperature period and changes in early gonadal development in yellowtail, including a transient difference in the initial appearance of SYCP3‐positive germ cells in males and reduced development of CN/PN‐stage oocytes in females. These findings suggest that light control may help maintain or improve growth performance during high‐temperature periods and may be associated with changes in early oocyte development in yellowtail. If practical shading conditions are established, shading may become a viable approach to support growth during high‐temperature periods while controlling reproductive progression.

## AUTHOR CONTRIBUTIONS

**Yuya Hasegawa:** Conceptualization; methodology; investigation; formal analysis; data curation; visualization; writing—original draft; writing—review and editing; project administration. **Kiko Shibata:** Resources; investigation; writing—review and editing. **Taiga Abe:** Resources; investigation. **Takurou Hotta:** Resources; investigation; funding acquisition; project administration; writing—review and editing.

## FUNDING INFORMATION

This study was supported in part by grants from the Japan Fisheries Research and Education Agency and was conducted in part as a commissioned project of the Japan Fisheries Agency to promote the growth and industrial development of aquaculture.

## CONFLICT OF INTEREST STATEMENT

The authors declare no conflicts of interest.

## Supporting information


**Figure S1.** Representative H&E‐stained sections of gonads. Gonads from males and females are shown at the initial time point (a, b). Gonads from males (left) and females (right) reared under control or continuous darkness (0L:24D) are shown at 2 weeks (c–d′) and 8 weeks (e–f′). Panels: initial male (a) and initial female (b); 2‐week male Control (c), 2‐week male 0L:24D (c'), 2‐week female Control (d) and 2‐week female 0L:24D (d′); 8‐week male Control (e), 8‐week male 0L:24D (e′), 8‐week female Control (f) and 8‐week female 0L:24D (f′). Scale bars, 50 μm.
**Figure S2.** Isotype‐control staining and corresponding immunostaining in male and female yellowtail gonads. All panels were obtained from control fish. Unless otherwise indicated, male and female gonads sampled at Week 8 were used. The male SYCP3, male Vasa (mouse) and male normal rabbit IgG panels were obtained from male gonads sampled at Week 10. Images show merged fluorescence of DAPI and the corresponding antibody channel. Antibody signals are green, and nuclei are counterstained with DAPI. Normal rabbit IgG and normal mouse IgG were used in place of rabbit derived and mouse‐derived primary antibodies, respectively, at the same concentration. Isotype control and corresponding primary‐antibody staining were performed using sections from the same individuals. Exposure settings for each antibody channel were identical between the isotype control and the corresponding primary‐antibody staining. Scale bars = 50 μm.
**Figure S3.** Multiple amino‐acid sequence alignment of SYCP3 in yellowtail and other vertebrates. Amino‐acid sequences of SYCP3 from human (Hs, *Homo sapiens*), mouse (Mm, *Mus musculus*), yellowtail (Sq, *Seriola quinqueradiata*), zebrafish (Dr, *Danio rerio*) and sterlet sturgeon (Ar, *Acipenser ruthenus*) were aligned to assess conservation of the C‐terminal region. Yellow boxes indicate the C‐terminal region examined as supporting information for the interpretation of SYCP3 immunostaining in yellowtail. Grey shading indicates conserved residues, and asterisks indicate residues identical among all aligned species.

**Table S1.** Selected published applications of the same commercial antibodies used in this study.

## Data Availability

The data that support the findings of this study are available from the corresponding author upon reasonable request.
