# Supplementary material for: Continuous darkness is associated with improved growth performance during a high‐temperature period and sex‐specific modulation of early gonadal development in juvenile yellowtail (Seriola quinqueradiata)
Source: J Fish Biol. 2026 Aug 14;109(4):2371–82. doi: 10.1111/jfb.70617 (PMC13675503; doi:10.1111/jfb.70617)
Supplement: Supplementary file 1 [file JFB-109-2371-s001.pdf]

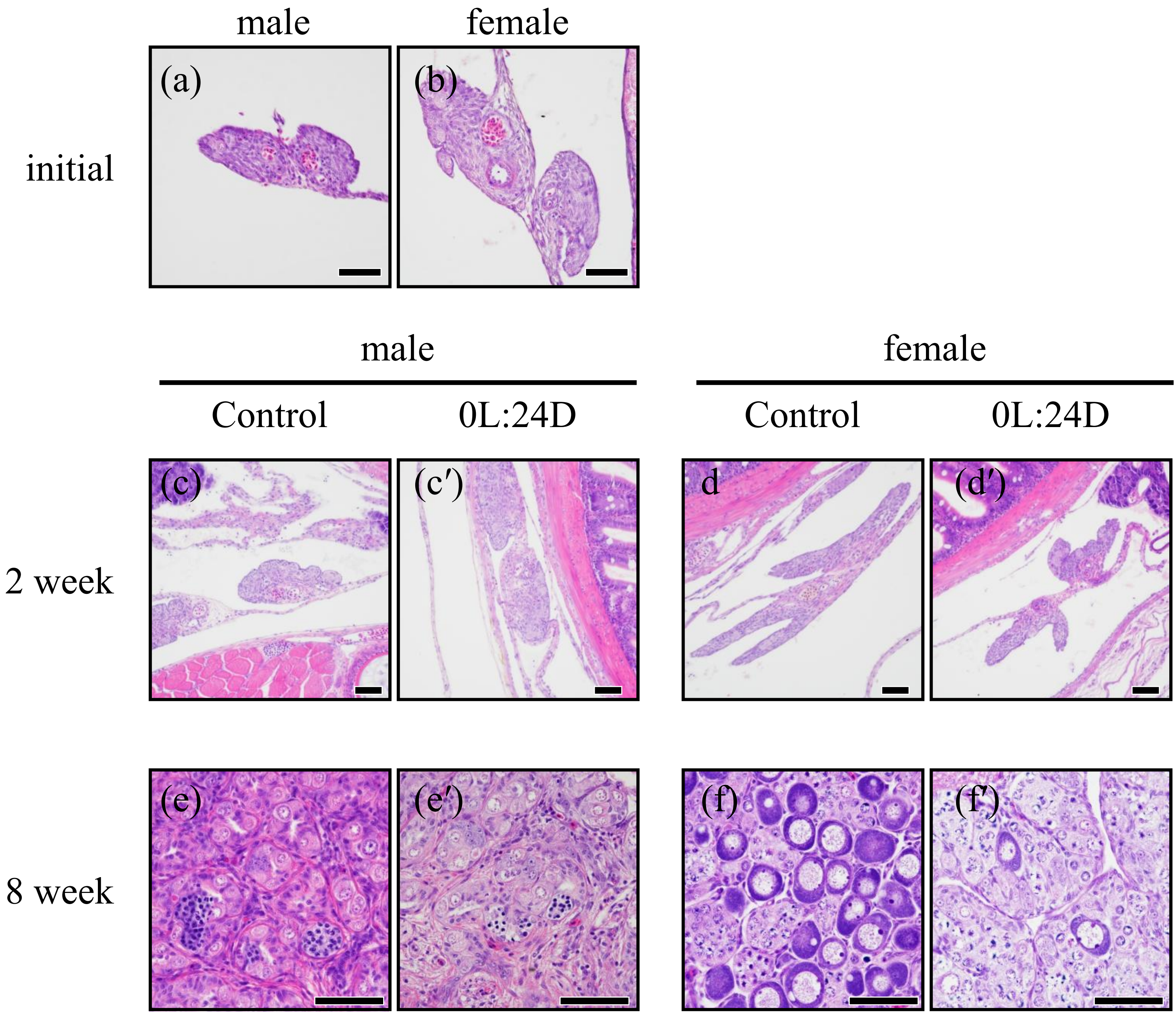

Supporting Information Figure S1. Representative H&E-stained sections of gonads. Gonads from males and females are shown at the initial time point (a, b). Gonads from males (left) and females (right) reared under control or continuous darkness (0L:24D) are shown at 2 weeks (c–d′) and 8 weeks (e–f′). Panels: initial male (a) and initial female (b); 2-week male Control (c), 2-week male 0L:24D (c′), 2-week female Control (d), and 2-week female 0L:24D (d′); 8-week male Control (e), 8-week male 0L:24D (e′), 8-week female Control (f), and 8-week female 0L:24D (f′). Scale bars, 50 μm.

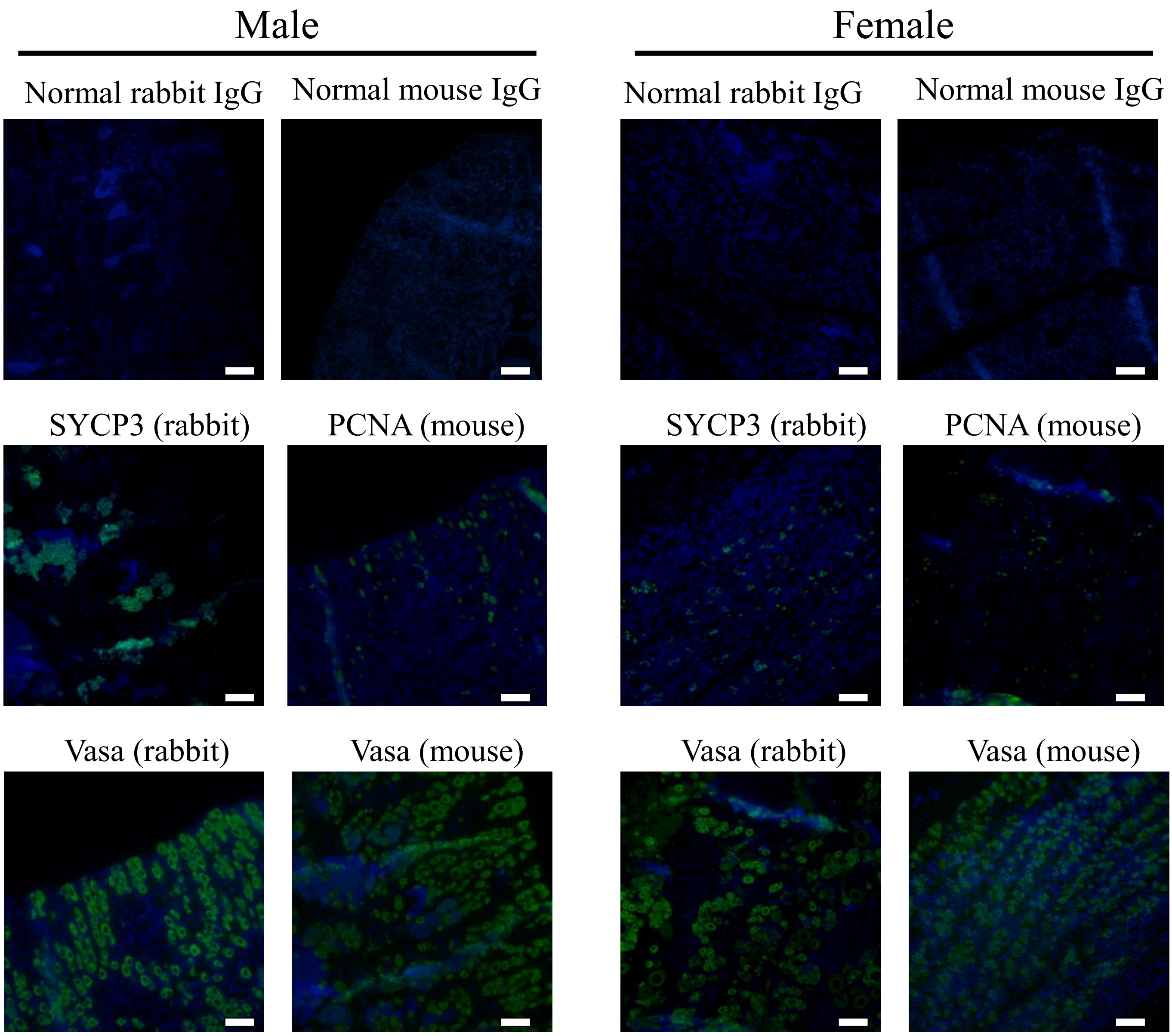

Supporting Information Figure S2. Isotype-control staining and corresponding immunostaining in male and female yellowtail gonads.

All panels were obtained from control fish. Unless otherwise indicated, male and female gonads sampled at week 8 were used. The male SYCP3, male Vasa (mouse), and male normal rabbit IgG panels were obtained from male gonads sampled at week 10. Images show merged fluorescence of DAPI and the corresponding antibody channel. Antibody signals are green, and nuclei are counterstained with DAPI. Normal rabbit IgG and normal mouse IgG were used in place of rabbit-derived and mouse-derived primary antibodies, respectively, at the same concentration. Isotype-control and corresponding primary-antibody staining were performed using sections from the same individuals. Exposure settings for each antibody channel were identical between the isotype control and the corresponding primary-antibody staining. Scale bars = 50 μm.

```
Hs   --------------------MVSSGKKYSRKSGKPSVEDQFTRAYDFE-T   29
Mm   MLRGCGDSDSSPEPLSKHLKMVPGGRKHSGKSGKPPLVDQPKKAFDFE-K   49
Sq   ------------MPVANQTGRMATGRKQMKKKHPEE--KSDKKVFDFIVE   35
Dr   --------------------MAAAGRKQNKKTKHTDGVVTDLKAFDFTVL   30
Ar   --------------------MAAAGRKQTSKTSKGAQQEVTDVPVYRFNE   30
                             * *   *

Hs   EDKKDLSGSEEDVIEGKTAVIEKRRKKRSSAGVVEDMGG--------EVQ   71
Mm   DDK-DLSGSEEDVADEKAPVIDKHGKKRS-AGIIEDVGG--------EVQ   90
Sq   DEKKELSGSEDEVREDETPIVDKLAKKRPAADFEAGEAT---CAVGNEVQ   82
Dr   EEKKES-GSDEDTRDDETPIIDKLSKKR-SADTFDDELN---SGVGNEVQ   75
Ar   DKTTGLSGSEDDIWEEETPVVEKHGKKRPVIMPIHDVHDDEEVNMGGEVH   80
            **                ***                   **

Hs   NMLEGVGVDINKALLAKRKRLEMYTKASLKTSNQKIEHVWKTQQDQRQKL  121
Mm   NMLEKFGADINKALLAKRKRIEMYTKASFKASNQKIEQIWKTQQEEIQKL  140
Sq   SMLEKFGADISKVMQAKKKRLECLTKNYMKGSQHKLEQLWNNYHSQRQKM  132
Dr   SMLERFGADISKAMQTKRKRLEVLTKNSLKGSTQKLEQMWKTQQNQRQKL  125
Ar   NMLEKFGADISKAMQAKRKRLEMYTKTSLKNSNQKLEKMWKTQQNQRQKL  130
      ***  * **      * ** *  **   * *  * *  *       **

Hs   NQEYSQQFLTLFQQWDLDMQKAEEQEEKILNMFRQQQKILQQSRIVQSQR  171
Mm   NNEYSQQFMNVLQQWELDIQKFEEQGEKLSNLFRQQQKIFQQSRIVQSQR  190
Sq   TQQYSQQVSTALQQWETEAQRAEEQEEKLNNLFRQQQKILQQARVVQNQK  182
Dr   TQDHSQQVFSVLQQWESDVLKSEEQEEKLNNLFRQQLKLFQQARVVQKQK  175
Ar   TEDYSQQFFSVLQQWEVDVQKSEEQEEKMTNLFRQQQKVFQQARIVQNQK  180
         ***     ***       *** **  * ****    **   ** *

Hs   LKTIKQLYEQFIKSMEELEKNHDNLLTGAQNEFKKEMAMLQKKIMMETQQ  221
Mm   MFAMKQIHEQFIKSLEDVEKNNDNLFTGTQSELKKEMAMLQKKVMMETQQ  240
Sq   LKTVRELYEQFVKNMEDMEKGHEAFLQGAQQELKKEMATLQKKILMDTQQ  232
Dr   IKTINDLHEQFVKNIEEMEKSHEAFLQGTQMELKKEMALLQKKIMMDTQQ  225
Ar   LKTIKQMYEQFLKNMEDMEKNQETFLMGAQSELKKEMAMLQNKIMMDTQQ  230
             *** *  *  **          * * **** ****  * ***

Hs   QEIASVRKSLQSMLF                                     271
Mm   QEMANVRKSLQSMLF                                     290
Sq   QEMATVRKSLQSMLF                                     282
Dr   QEMATVRKSLQSMLF                                     275
Ar   QEMATVRKSLQSMLF                                     280
     ** * **********
```

Supporting Information Figure S3. Multiple amino-acid sequence alignment of SYCP3 in yellowtail and other vertebrates.

Amino-acid sequences of SYCP3 from human (Hs, *Homo sapiens*), mouse (Mm, *Mus musculus*), yellowtail (Sq, *Seriola quinqueradiata*), zebrafish (Dr, *Danio rerio*), and sterlet sturgeon (Ar, *Acipenser ruthenus*) were aligned to assess conservation of the C-terminal region. Yellow boxes indicate the C-terminal region examined as supporting information for the interpretation of SYCP3 immunostaining in yellowtail. Gray shading indicates conserved residues, and asterisks indicate residues identical among all aligned species.
