# Supplementary material for: Continuous darkness is associated with improved growth performance during a high‐temperature period and sex‐specific modulation of early gonadal development in juvenile yellowtail (Seriola quinqueradiata)
Source: J Fish Biol. 2026 Aug 14;109(4):2371–82. doi: 10.1111/jfb.70617 (PMC13675503; doi:10.1111/jfb.70617)
Supplement: Supplementary file 2 [file JFB-109-2371-s002.pdf]

Supporting Information Table S1.
Selected published applications of the same commercial antibodies used in this study.

Marker	Host	Catalog no.	Species	Application	Reference
SYCP3	Rabbit	Abcam ab15093	Zebrafish (*Danio rerio*)	IHC	Annus et al. 2022
			Guppy (*Poecilia reticulata*)	Chromosome spread IF	Lisachov et al. 2015
			Loricariid catfish (*Hypancistrus zebra*)	Chromosome spread IF	Almeida et al. 2023
			Mouse (*Mus musculus*)	WB and IHC	Dong et al. 2021
Vasa/DDX4	Rabbit	GeneTex GTX128306	Zebrafish (*Danio rerio*)	WB	Krishnakumar et al. 2018
			Honmoroko (*Gnathopogon caerulescens*)	IHC	Higaki et al. 2017
PCNA	Mouse	Abcam ab29	Gilthead seabream (*Sparus aurata*)	IHC	Pavón-Muñoz et al. 2016

Abbreviations: IF, immunofluorescence; IHC, immunohistochemistry; SC, synaptonemal complex; WB, Western blot. “Application” indicates the application reported in the cited publication. SC spread IF was included because it provides published evidence that the same antibody detects SYCP3-associated meiotic chromosome structures, although it does not constitute direct validation in yellowtail tissue sections.
